# Artificial intelligence for biomarker prediction in gastric cancer: from histopathology to multimodal integration

**DOI:** 10.3389/fonc.2026.1845038

**Published:** 2026-06-16

**Authors:** Yesul Jeong, Sangjeong Ahn, Sung Hak Lee

**Affiliations:** 1 Department of Hospital Pathology, St. Vincent’s Hospital, College of Medicine, The Catholic University of Korea, Seoul, Republic of Korea; 2 Department of Pathology, College of Medicine, Korea University, Seoul, Republic of Korea; 3 Department of Biomedical Informatics, College of Medicine, Korea University, Seoul, Republic of Korea; 4 Department of Hospital Pathology, Seoul St. Mary’s Hospital, College of Medicine, The Catholic University of Korea, Seoul, Republic of Korea

**Keywords:** artificial intelligence, biomarker, digital pathology, gastric carcinoma, multimodal integration, precision medicine, whole-slide imaging

## Abstract

**Introduction:**

Gastric cancer (GC) exhibits substantial molecular heterogeneity, necessitating precision oncology approaches. Conventional biomarker assessments, including immunohistochemistry, *in situ* hybridization, polymerase chain reaction, and next-generation sequencing, are the diagnostic standard but resource-intensive and may be limited by tissue availability, cost, and turnaround time. Artificial intelligence (AI)–enabled computational pathology using whole-slide images (WSIs) has emerged as a scalable approach for inferring molecular and immune phenotypes directly from routine hematoxylin and eosin–stained histopathological images.

**Methods:**

This review synthesizes recent advances in AI-based WSI analysis for biomarker assessment in GC, focusing on molecular subtype prediction, actionable genetic alterations, immune-related features, tumor microenvironment characterization, and multimodal integration.

**Results:**

AI models have demonstrated promising performance in predicting microsatellite instability and Epstein–Barr virus status, supporting their potential use as prescreening or triage tools to prioritize confirmatory testing. In addition, these approaches enable the quantitative characterization of the tumor microenvironment by mapping tertiary lymphoid structures and immune architecture, providing prognostic insights. Multimodal integration of histopathology with radiologic, genomic, and clinical data has shown improved predictive performance compared to single-modality approaches, particularly for recurrence and treatment responses.

**Discussion:**

However, challenges remain, including model interpretability, variability in performance across different datasets, and incomplete data across modalities. Future directions include prospective multicenter validation in clinical workflows, standardization of evaluation frameworks, and implementation of uncertainty estimation to support clinical decision-making. Overall, AI–enabled digital pathology represents a promising approach for advancing precision oncology in GC by improving biomarker assessment and providing insights into tumor biology.

## 1 Introduction

Gastric cancer (GC) continues to be a significant global health burden, ranking among the most frequently diagnosed cancers and leading causes of cancer-related deaths worldwide, with approximately one million new cases identified annually (1). GC exhibits substantial molecular heterogeneity, with distinct subtypes characterized by unique genomic alterations, immune microenvironments, and clinical behaviors (2). This complexity necessitates precision medicine approaches that tailor therapeutic strategies to individual patients based on clinically relevant molecular biomarkers (2, 3).

Traditional biomarker assessment in GC relies on immunohistochemistry (IHC), *in situ* hybridization (ISH), and molecular profiling techniques such as polymerase chain reaction (PCR) and next-generation sequencing (NGS). Although these methods provide definitive molecular characterization, they require additional tissue sections, specialized reagents, technical expertise, and considerable time and cost. Tissue availability may be limited, particularly for biopsy specimens, and molecular testing may not be universally accessible in resource-limited settings (3). Moreover, the growing demand for integrated biomarker reporting has increased the workload and interpretive complexity of routine pathology practices (4).

Diagnostic pathology is undergoing a paradigm shift from microscope-based evaluation of glass slides to digital pathology, enabled by whole-slide imaging (WSI) (5, 6). The digitization of hematoxylin and eosin (H&E)-stained slides has accelerated research into artificial intelligence (AI) for computational pathology and biomarker inference directly from histopathology (7, 8). Deep learning models, especially convolutional neural networks (CNNs) and vision transformers, can identify complex morphological patterns that correlate with underlying molecular alterations, enabling rapid and cost-effective biomarker inference from standard histopathology (9, 10). Beyond single-modality histology analysis, multimodal approaches that integrate WSIs with genomic, transcriptomic, radiomic, and clinical data may capture complementary information and improve predictive accuracy (11).

Recent reviews have broadly summarized the applications of AI in GC management, including endoscopy, imaging, surgery, treatment response prediction, multimodal decision support, and prognosis modeling (10). However, the specific question of how routine histopathology can be transformed into biomarker-oriented decision support remains less explicitly addressed. In contrast to previous reviews, the present review focuses on H&E-based and pathology-centered biomarker prediction, including microsatellite instability (MSI), Epstein–Barr virus–positive (EBV), human epidermal growth factor receptor 2 (HER2) status, programmed death-ligand 1 (PD-L1) expression, tumor mutational burden (TMB), tumor microenvironment (TME) architecture, and immune-signature subtypes, as well as their integration into pathology workflows.

The central message is that AI in GC is moving from generic image classification to biomarker-oriented, histology-grounded decision support. At present, the most clinically plausible role of these models is not to replace established IHC, ISH, PCR, or NGS assays but to support prescreening, triage, risk enrichment, spatial interpretation, and prioritization of confirmatory testing. We summarize the current landscape of AI-enabled WSI analysis for biomarker assessment (**Table 1**) and multimodal integration (**Table 2**) in GC, emphasizing its role in precision medicine and highlighting the methodological, regulatory, and workflow-related challenges that must be addressed for real-world clinical implementation.

**Table 1:** Current landscape of AI-enabled WSI analysis for biomarker assessment in gastric cancer.

Author	Year	Architecture	Task endpoints	Training/validation dataset (n)	External validation dataset (n)	Performance	Performance of External test
Kather et al. (12)	2019	ResNet18	MSI	315 patients, TCGA	185 patients	AUC 0.81	AUC 0.69
Kather et al. (7)	2020	ShuffleNet	Molecular alteration	321 WSIs, 3-fold patient-level CV	NA	AUC for the top 8 mutations ranged from 0.66 to 0.78	NA
Valieris et al. (13)	2020	ResNet34, RNN	MSI, MLH1, TMB	394 WSIs of 369 patients, TCGA Train/Val: 70%/30%	NA	AUC 0.81	NA
Hinata & Ushiku (14)	2021	VGG16	MSI & EBV	408 cases (Train: 326; 4-fold CV within training; Test: 82)	244, TCGA (Train: 48; Predict: 196)	AUC 0.947	AUC 0.870
Huang et al. (15)	2021	GastroMIL (RegNet, RNN), MIL-GC (MLP)	Histology, Prognosis (OS risk prediction)	639 WSIs (Train: 443; Val: 196)	91 WSIs	Train: C-index 0.728; Val: C-index 0.671	C-index 0.657; Multivariable HR 1.803
Jang et al. (16)	2021	Inception-v3	Mutations (CDH1/ERBB2/KRAS/PIK3CA/TP53)	745 WSIs of 302 patients (one among 5 mutated genes); 183 patients (wild-type of 5 genes); 10-fold CV, TCGA (frozen/FFPE)	96 patients (FFPE)	AUC of CDH1/ERBB2/KRAS/PIK3CA/TP53 0.842/0.751/0.793/0.862/0.727 (Frozen) 0.781/0.661/0.858/0.828/0.727 (FFPE)	AUC of CDH1/ERBB2/KRAS/PIK3CA/TP53 0.778/0.833/0.838/0.761/0.775
Muti et al. (17)	2021	ShuffleNet	MSI, EBV	2823 patients (known MSI status); 2685 patients (known EBV status); Ten cohorts pooled across seven countries; Train: 5 largest cohorts (BERN/CLASSIC/MAGIC/LEEDS/TCGA)	Val: remaining cohorts (KCCH/AUGSB/ITALIAN/KOELN/TUM)	Train: AUC 0.810 for EBV; AUC 0.761 for MSI	Val: AUC 0.863 for MSI; AUC 0.859 for EBV (highest AUC among several external datasets)
Wang et al. (18)	2021	U-Net, ResNet-50	T/MLN (LN metastasis detection and prognosis stratification) Histology, Prognosis	6418 slides of 857 patients, including 15,362 LNs; Test: (two cohorts) 2044 WSIs of 215 patients 904 WSIs of 92 patients	NA	Accu 96.9% Sens 98.5% Spec 96.1%	T/MLN as cancer-specific survival (multivariable HR 1.39)
Zhang et al. (19)	2021	ResNet18	TME, EBV, Prognosis	400 WSIs of 375 patients (Train/Test: 70%/30%)	NA	AUC 0.85 for tumor tiles; AUC 0.81 for normal tiles	NA
Ba et al. (20)	2022	DeepLab v3	Histology, benign vs. malignancy (Human-AI assisted)	110 WSIs (gastric biopsy)	NA	AUC 0.911 vs 0.863 (P = 0.003); Sens 90.63% vs 82.75% (P = 0.010); Spec NS; 22.68s vs 26.37s (P = 0.033) (with AI vs without AI)	NA
Flinner et al. (21)	2022	DenseNet161 + bagging ensemble	TCGA classification (EBV/MSI/GS/CIN)	133 WSIs, TCGA	65 WSIs, UKC	AUC around 0.76	NA
Jeong et al. (22)	2022	EBVNet (ResNet50+InceptionV3)	EBV	Patch-wise: 319 WSIs Fine-tuning: 108 WSIs, TCGA	60 WSIs	AUPRC of EBVNet/Pathologists: 0.65/0.41	NPV/Sens/Spec/Prec/F1: 0.98/0.86/0.92/0.60/0.71
Ghaffari et al. (23)	2022	ResNet/EfficienNet/ViTvs. MIL/AttMIL/CALM	MSI, EBV	302 patients (MSI), 304 patients (EBV); 3-fold patient-level CV	327, TCGA	Highest performance: AUC 0.761 for MSI (ResNet); AUC 0.853 for EBV (EfficientNet)	MSI AUC 0.739 (EfficientNet); EBV 0.813 (CLAM)
Pomponio et al. (24)	2022	Handcrafted image feature extraction with machine learning	TME, Immune: (inflamed/excluded/desert) by CD8+ TIL from multiplex IF WSIs	110 cases (5 cancer types); 103 cases (multiplex-image analysis); Train: 68; Test: 35 (split by cancer types for generalizability)	NA	74% concordance with pathologist immune-phenotype assignment; AUC >0.85 for key binary separations (e.g., inflamed vs non-inflamed; excluded vs desert)	NA
Su et al. (25)	2022	ResNet18	MSI	467 patients (divided into 3 cohorts); Train: 348 WSIs (80%); Val: 12.5% of training tiles; Test: 88 WSIs (20%)	Integrating test: 31 WISs	Accu 86.36%	Integrated end-to-end system: Accu 83.87%
Wang et al. (26)	2022	DEMoS EfficientNet-b1 ensemble	TCGA classification (EBV/MSI/GS/CIN)	295 patients of 332 WSIs, TCGA; 10-fold CV within 80% Test: 20% patients	NA	Patient AUC for GS/EBV/CIN/MSI: 0.897/0.764/0.890/0.898	NA
Zheng et al. (27)	2022	ResNet50+EBVNet uncertainty-weighted human–AI fusion	EBV	1006 WSIs of 727 patients; 5-fold internal CV	417 patients and WSIs, MultiCenter; 258 WSIs of 239 patients, TCGA	AUROC 0.969	AUC 0.941 (MultiCenter), AUC 0.895 (TCGA); AUC up to 0.969 (MultiCenter), AUC up to 0.939 (TCGA) using fusion
Lee et al. (28)	2023	Inception v3	MSI	331 patients, TCGA (frozen+FFPE); 10-fold patient-level CV	383 patients (FFPE)	AUC 0.893 (TCGA frozen); AUC 0.902 (TCGA FFPE)	AUC 0.874 (SSMH); using TCGA FFPE trained model)
Li et al. (29)	2023	ResNet18, Mask R-CNN, CART	Immune (Tertiary Lymphoid Structures)	1924 patients (GI cancer from 7 cohorts)	NA	NA	Accu 95.7–97.7%; TLS score stratifies OS: High vs Low HR 0.27; Low vs None HR 0.65
Saldanha et al. (30)	2023	RetCCL, Swarm learning	MSI, EBV	418/903/601 (from 3 multicohorts)	443, TCGA	NA	AUC ~0.809 for MSI; AUC ~0.837 for EBV
Veldhuizen et al. (31)	2023	attMIL	Laurén classification	166, TCGA; five-fold cross-validation	322, European; 243, Japanese	AUC 93% with a lower SD of 86%	HR 1.46 (1.18-1.65); HR 1.41 (1.20-1.57)
Wei et al. (32)	2023	DeepCox-SC: InceptionResNetV2	Histology, Prognosis	382 WSIs of 357 patients, TCGA; Ten-fold cross-validation	30 patients, Ruijin	C-index 0.660 ± 0.057	C-index 0.657; Risk score (external HR 2.912)
Yu et al. (33)	2023	RA-NL	MSI	448 WSIs (Train/Val: 8:2); Train: 361 WSIs; Val: 87 WSIs	78,189 MSI and 140,391 MSS patches, TCGA; Train/Val/Test: 70%, 15%, 15%	Slide-wise Accu 91.95%	Accu 96.53%, AUC 0.99 (transfer learning)
Han et al. (34)	2024	Pathomic (Otsu, PyRadiomics, GBM)	Immune (predict activated CD4 memory T-cell infiltration, high vs low)	Train: 165 cases, TCGA (70%); Val: 69 cases, TCGA (30%)	NA	Train: AUC 0.844; Val: AUC 0.750 OS (HR = 0.506)	NA
Reddy et al. (35)	2024	CNN	Histology (Stage I vs. II)	3540, WSIs; Train: 2832 WSIs (80%); Val: 354 WSIs (10%); Test: 354 WSIs (10%)	NA	Accu/Prec 100%/100%; Sens/Spec/F1 97.09%/100%/98.31%; AUC 0.999	NA
Tian et al. (36)	2024	DeepRisk multi-scale attention-based MIL, DPS	DPS (prognosis prediction using OS, DFS); TME association with neoadjuvant chemotherapy response	1,120 patients; 82 patients received neoadjuvant chemotherapy	536 WSIs of 268 patients, TCGA; 277 patients, SOBC	C-index at 5×/10×/20×: 0.781/0.812/0.821; Multi-scale 5×+10×+20×: C-index 0.828; DPS C-index: 0.84 for OS, 0.71 for DFS	C-index 0.74 for OS, TCGA; C-index 0.67 for OS, SOBC
Hong et al. (37)	2025	TMEPATH AG-GAN + EfficientNet-B3	TMEpath (TSR+TIL) TME, prognosis prediction	320 patients	186 patients	HR 1.768 for high vs. low-risk; C-index 0.545 for OS; AUC 0.561 for RFS	HR 2.435 for high vs. low-risk; C-index 0.579 for OS; AUC 0.587 for RFS
Liao et al. (38)	2025	HER2Net (SegNet+ResNet50+RF)	HER2	646 WSIs; Train: 531 WSIs of 520 patients; Val: 115 WSIs of 111 patients	102 WSIs of 101 patients (multi-center)	Accu 0.9043; PPV 0.6400; Sens 0.8889	Accu 0.8922; PPV 0.6923; Sens 0.8571
Park et al. (39)	2025	MSI-SEER (DGP-based with WSL)	MSI, immunotherapy response prediction	Test+Val: 1101 WSIs (from 12 multicenter/multiethnic datasets)	NA	Precision/Recall/F1 0.493/0.698/0.578 (original performance for MSI)	Precision/Recall/F1 0.614/0.628/0.621 (after most uncertain predictions removed)
Wu et al. (40)	2025	Hist2ST, ConvMixer, Transformer encoder	Immune, Prognosis, TLS	253 WSIs	15 patients	Accu 0.977	Mean Accu 0.924

Accu, accuracy; AUC, area under the receiver operating characteristic curve; AUROC, area under the receiver operating characteristic curve; AUPRC, area under the precision-recall curve; BER/CLASSIC/MAGIC/LEEDS/TCGA/KCCH/AUGSB/ITALIAN/KOELN/TUM/SSMH/SOBC, patient cohort names; C-index, concordance index; CD8+ TIL, CD8-positive tumor-infiltrating lymphocytes; CIN, chromosomal instability; CV, cross-validation; DFS, disease-free survival; DGP, deep generative pretraining; DPS, digital pathology signature; EBV, Epstein-Barr virus; EfficientNet, convolutional neural network architecture; FFPE, formalin-fixed, paraffin-embedded; GBM, gradient boosting machine; GS, genomically stable; HR, hazard ratio; IF, immunofluorescence; LN, lymph node; MSS, microsatellite stable; MSI, microsatellite instability; MIL, multiple instance learning; MLH1, MutL homolog 1; MLN, metastatic lymph node; MSI-SEER, MSI prediction model using SEER data (DGP based with WSL); NA, not available; NPV, negative predictive value; OS, overall survival; PPV, positive predictive value; RFS, recurrence-free survival; ResNet, residual neural network; SOBC, stomach cancer cohort (external); Sens, sensitivity; Spec, specificity; SSL/WSL, semi-supervised/weakly supervised learning; TCGA, The Cancer Genome Atlas; TLS, tertiary lymphoid structures; TME, tumor microenvironment; TSR, tumor-stroma ratio; Val, validation; WSI, whole-slide image.

**Table 2:** Multimodal approaches in gastric cancer.

Author	Year	Architecture	Task	Multimodal method	Cohort dataset	Performance
Guo et al. (41)	2023	AttMIL-MM, ResNet-101	Preoperative prediction of lymph node metastasis	Histology slides (multi-scale), clinical data	309 patients (radical surgery), 157 patients (biopsy); External 306, TCGA	Adding clinical data to H&E improves AUC at each scale (e.g., 20×: 0.869 vs 0.832), multi-scale fusion further improves over any single scale (0.877 vs 0.864–0.869). External TCGA performance drops (~0.69 AUC), highlighting stain/scanner/domain variability as a key limitation.
Wei et al.^a^	2023	MultiDeepCox-SC: Cox fusion of DeepCox-SC risk + age + 10-gene expression (SIS→LASSO)	OS risk prediction, prognostic stratification	Image-derived risk + clinical age + 10 gene expression	382 WSIs of 357 patients, TCGA; Ten-fold cross-validation; External cohort: Ruijin 30 patients	OS prediction C-index: histopathology only (0.660); histopathology + clinical + gene expression (0.744)
Gao et al. (42)	2024	iSCLM CT (ResNet-34) + Biopsy WSI (ResNet-18 feature + Graph Attention Network) + supervised contrastive cross-modal alignment	Predict neoadjuvant chemotherapy response in GC using TRG (surgical) and RECIST (non-surgical)	Contrast-enhanced CT (primary tumor and lymph node ROI) + endoscopic biopsy H&E WSI (pathologist-annotated ROI)	10-center retrospective cohort n=2,387 (development 1,208; incremental 795; external test 1:162; external test 2:222) + prospective test n=132	CT-only AUC 0.802/0.822/0.781 (external1/external2/prospective); Pathology-only AUC 0.550/0.596/0.546; iSCLM 0.866/0.876/0.846
Chen et al. (43)	2024	Multi-Modal Model (MuMo)	Predict treatment response in patients with anti-HER2 GC	Clinical information, pathology, radiology	Internal: n=390; Anti-HER2: n=271; Anti-HER2 + immunotherapy: n=119; External cohort: n=39	Radi-only AUC 0.639; Path-only AUC 0.703; HER2+ AUC 0.821; HER2+ICI AUC 0.914; External (HER2+) AUC 0.884
Li et al. (44)	2024	ResNet 18 (TMB) multimodal fusion model (MCB/CBP)	TMB high/low classification	WSI, clinical, multi-omics	N=326, TCGA	Absolute +0.222; relative +29.6% (from reported AUCs) Multimodal fusion improves over pathology-only (image+mRNA AUC 0.971 vs image-only 0.749). Selecting specific omics (especially mRNA) outperforms “all-omics together,” highlighting the importance of feature selection and modality choice.
Xie et al. (45)	2024	ResNet-50, cross-modal co-attention, transformer encoders + attention pooling	Multitask prediction in GC using pathology + transcriptomics: Survival prediction (time-to-event risk; Harrell’s concordance index) Pathological stage classification (early stage I–II vs advanced stage III–IV) Lymph node status classification (node-positive vs node-negative).	WSI, gene expression (231 genes from 9 oncology pathways)	95 patients (846 WSIs) Five repeated stratified splits (train/validation/test = 3:1:1)	Multimodal improves consistently: survival C-index improves from 0.564 (CLAM) to 0.613 (GaCaMML); stage and lymph node classification show double-digit accuracy gains
Gao et al. (46)	2025	Multi-step Multi-modality Ensemble Survival (MMES) model	Overall survival risk prediction/ prognostic stratification in GC using histology + immune spatial information (+ clinical covariates)	H&E-stained images/multiplex immunohistochemistry (CD68, CD163, CD8, FOXP3, PD-L1, panCK) + clinical data (age, sex, grade; stage in TCGA extension)	Multi-center TMA cohort n=456 across 5 batches; temporal split training n=268, test1 n=90, test2 n=98 (all-test n=188). TCGA-STAD n=354 patients (379 WSIs)	Multimodal outperformed single-modality models (+5.4% C-index) and end-to-end multimodal baselines (C-index 0.563–0.569) with 9.9% average C-index improvement
Huang et al. (47)	2025	Radiopathomic signature (RPS)	Predict treatment response and stratify prognostic risk in GC	CT + WSIs + clinicopathologic biomarkers (PD-L1, MSI-H, EBV, HER2)	298 patients from 3 centers	AUCs of 0.978/0.863/0.822 in the training/internal/external validation cohorts, respectively; outperformed conventional biomarkers (e.g., CPS, MSIH, EBV, HER-2, TNM)
Mao et al. (48)	2025	MultiDeepsurv GLFUnet, GCN, Sfusion	Prognostic accuracy for survival prediction	WSI, gene expression and clinical data	TCGA 442 WSIs of 416 patients; Five-fold cross-validation	Pathology only: C-index 0.724, AUC 0.759 Gene expression only: C-index 0.600, AUC 0.649 Clinical only: C-index 0.783, AUC 0.814 Multimodal: C-index 0.806, AUC 0.842
Ding et al. (11)	2025	RSA multimodal recurrence model Multi-step ensemble survival modeling	Early postoperative recurrence prediction, prognostic stratification in locally advanced GC	Pathomics, radiomics, clinical, transcriptomics (for analysis)	N=1,580 LAGC patients from six centers; Training: n=770; Internal validation: n=362; External validation: n=448 Prospective trial-derived validation (multicenter): n=257 (primary surgery n=93; neoadjuvant XELOX n=86; neoadjuvant DOX n=78). Public cohort (The Cancer Imaging Archive): n=41. Prospective RNA seq cohort, n=133.	Clinical-only: AUC 0.756 Radiomics-only: 0.806 Pathomics-only: 0.82 RSA (clinical + radiomics + pathomics): Training: AUC 0.903 Internal/External validation: 0.902/0.884-0.896 Prospective trial-derived cohort: surgery-only subgroup 0.889; neoadjuvant subgroup 0.736; Public cohort 0.898

AUC, area under the receiver operating characteristic curve; CLAM, Clustering-constrained Attention Multiple-instance learning; CT, computed tomography; EBV, Epstein–Barr virus; GC, gastric cancer; HER2, human epidermal growth factor receptor 2; ICI, immune checkpoint inhibitor; H&E, hematoxylin and eosin; LAGC, locally advanced gastric cancer; LASSO, least absolute shrinkage and selection operator; mIHC, multiplex immunohistochemistry; MSI, microsatellite instability; NA, not available; OS, overall survival; PT/ROI, primary tumor/region of interest; RNA-seq, RNA sequencing; SIS, Sure Independence Screening; TCGA, The Cancer Genome Atlas; TCGA-STAD, TCGA stomach adenocarcinoma cohort; TLS, tertiary lymphoid structure; TRG, tumor regression grade; WSI, whole-slide image; XGBoost/GLFUnet/GCN/Sfusion, model architectures as specified in references. ^a^This study is also listed in **Table 1** because it covers topics relevant to both tables.

## 2 Diagnostic support and histologic subtyping

AI-assisted diagnostic tools have demonstrated high sensitivity in distinguishing benign and malignant lesions on biopsy WSIs. Multireader studies indicate that heatmap assistance improves sensitivity while significantly reducing review time, supporting a pragmatic “second-reader” role in routine workflows (area under the curve [AUC] 0.911 vs. 0.863; sensitivity 90.63% vs. 82.75%; mean review time per WSI 22.68 s vs. 26.37 s) (20).

Beyond binary detection, deep learning has been applied to standardize histologic subtyping. Using attention-based multiple-instance learning (MIL), classifiers for Laurén subtyping were trained on The Cancer Genome Atlas (TCGA; n = 166) with expert ground truth and externally validated in European (n = 322) and Japanese (n = 243) cohorts, achieving strong discrimination (area under the receiver operating characteristic curve [AUROC] 0.93 ± 0.07 (internal five-fold cross-validation) and improved survival stratification compared with pathologist-assigned Laurén classification (overall survival hazard ratios: 1.41 in Europe and 1.46 in Japan) (31). Similarly, AI models for detecting diffuse-type adenocarcinoma have achieved near-perfect performance (AUC 0.95–0.99) across multiple independent test sets by learning robust morphological features characteristic of diffuse-type GC (49).

In resected lymph nodes, AI pipelines can segment nodes, detect metastatic regions, and compute quantitative burden metrics, such as the tumor-to-metastatic lymph node area ratio (T/MLN), providing a continuous and interpretable measure of metastatic burden. In multivariate survival analyses, T/MLN remained independently associated with outcomes beyond nodal stage and improved prognostic discrimination compared with nodal staging alone. This approach is particularly attractive for routine implementation because lymph node slides are already generated for standard staging, and the output is transparent and clinically interpretable. Furthermore, AI-supported subtyping and automated lymph node analysis may directly influence treatment decisions by refining risk stratification and identifying subtle metastatic patterns, such as micrometastases (<2 mm), which are frequently overlooked during manual inspection. These frameworks allow for more precise N-staging and tailored therapeutic strategies for patients who might otherwise share the same clinical stage but have disparate outcomes. However, performance may be influenced by technical and biological heterogeneity, including specific histological subtypes, such as signet ring cell carcinoma, which can be more challenging for algorithms to identify (18).

## 3 Molecular subtyping: replicating the TCGA taxonomy

GC exhibits substantial inter-patient heterogeneity in etiologic drivers, histologic phenotypes, and therapeutic vulnerabilities, limiting the clinical utility of morphology-based stratification alone. To address this, TCGA performed multi-platform molecular profiling of primary, treatment-naïve GCs and proposed four molecular subtypes: EBV, MSI, genomically stable (GS), and chromosomal instability (CIN), derived from integrative clustering and translated into a pragmatic decision tree framework. TCGA taxonomy has been widely adopted for biological interpretation and clinical trial stratification (2). Within computational pathology, an emerging goal is to recover TCGA-like molecular subtypes directly from routine H&E slides as a scalable screening method. Wang et al. developed DEMoS, an ensemble unsupervised approach for four-class TCGA subtype prediction, reporting patient-level AUROCs of 0.897 (GS), 0.764 (EBV), 0.890 (CIN), and 0.898 (MSI) on an independent TCGA test split (26).

In parallel, clinical workflows often rely on pragmatic surrogate testing, such as EBV-encoded RNA *in situ* hybridization (EBER ISH), to establish EBV positivity and mismatch repair IHC (MLH1, PMS2, MSH2, and MSH6) to identify mismatch repair deficiency, as these assays are standardized and feasible (17).

### 3.1 MSI

MSI-associated GCs tend to respond better to immune checkpoint inhibitors because defective mismatch repair increases the mutational or neoantigen burden and frequently produces an immune-active microenvironment that is susceptible to programmed cell death protein 1 (PD-1) or PD-L1 blockade. In clinical practice, MSI is an established predictive biomarker for pembrolizumab in a tissue-agnostic manner and is recommended for routine testing in GC to guide immunotherapy selection (50).

MSI prediction from H&E-stained slides is one of the most extensively evaluated biomarker applications in gastric computational pathology. Kather et al. established a proof-of-concept for gastrointestinal cancers using a two-stage ResNet18 pipeline (tumor detection followed by MSI classification), achieving a patient-level AUROC of 0.81 in TCGA-STAD (n=315) (12). The study further supported biological plausibility by correlating image-derived MSI predictions with immune-related expression patterns, suggesting that deep learning models may capture the histologic correlates of the immune-active phenotype associated with mismatch repair deficiency (12). Subsequent studies have aimed to improve automation, interpretability, and external validity. Lee et al. reported a fully automated, sequential MSI classifier evaluated across TCGA frozen and formalin-fixed paraffin-embedded (FFPE) slides and an independent Korean FFPE cohort, with AUROCs of 0.893 (TCGA frozen), 0.902 (TCGA FFPE), and 0.874 (external Seoul St. Mary’s hospital FFPE). Notably, the performance further improved when the TCGA and Korean FFPE datasets were combined for training, supporting the value of dataset expansion and cross-population learning (28).

Other studies have focused on interpretability and workflow simulation. Su et al. proposed an interpretable end-to-end workflow linking histologic attribute recognition (tumor differentiation) with MSI prediction and demonstrated “integration testing” on unannotated WSIs to approximate deployment without tumor contours (25). However, this study also illustrates a recurring limitation: MSI labels were assigned at the patient level rather than at the tile level, meaning that intratumoral heterogeneity could introduce label noise during tile-level training. Yu et al. further explored architectural refinements by combining residual attention networks with non-local modules and visual context fusion to capture both fine-grained histologic detail and longer-range spatial dependencies across patches (33).

Large-scale multicenter studies have clarified the importance of cohort diversity and external validation. Muti et al. developed deep learning classifiers for MSI and EBV status using GC H&E slides from ten international cohorts across seven countries and showed that MSI prediction performance varied substantially across cohorts. In within-cohort cross-validation, MSI AUROCs ranged from 0.597 to 0.836, whereas pooled training followed by external validation yielded AUROCs ranging from 0.723 to 0.863 (17). This study is particularly informative because it demonstrates that the critical translational issue is not merely whether MSI can be predicted from H&E-stained slides, but whether the performance remains stable across different institutions, scanners, tissue-processing protocols, patient populations, and specimen characteristics. The observation that pooled international training improved robustness supports the need for diverse multicenter training sets rather than single cohort optimization alone.

Benchmarking studies further suggest that model architecture alone does not determine clinical utility. Ghaffari Laleh et al. systematically compared classical weakly supervised pipelines and MIL-based approaches across several whole-slide classification tasks, including gastric MSI prediction. In GC, the overall performance was lower than that in colorectal cancer, and no single method was consistently superior in external validation. More broadly, classical weakly supervised pipelines often performed as well as or better than more complex MIL-based approaches for mutation prediction tasks, emphasizing that preprocessing, weak-supervision strategy, cohort composition, and validation design may be as important as architectural novelty (23). Privacy-preserving multicenter learning has also been explored as a strategy to overcome the practical barriers to data sharing. Saldanha et al. applied swarm learning, a decentralized training framework, to MSI and EBV prediction in GC using three groups as physically separated training cohorts and TCGA as an external validation cohort. For MSI prediction, the swarm learning model achieved an AUROC of approximately 0.81, which was comparable to that of centralized training on pooled data (30). This study suggests that collaborative model development may be feasible without the direct exchange of raw patient-level pathology data, which is particularly relevant for GC, as robust AI development requires geographically and technically diverse datasets.

Collectively, MSI prediction is currently one of the most plausible early applications of AI-based biomarker inference in GC because it has a clear biological basis, established clinical actionability, and partially recognizable morphological and immune microenvironmental correlations. Nevertheless, current models should be positioned as high-sensitivity prescreening or prioritization tools for confirmatory IHC, PCR, or NGS.

### 3.2 EBV

Among the four molecular subtypes of GC, EBV-positive tumors have attracted particular attention because of their immune-related features and potentially targetable alterations. EBV-positive GCs are enriched with frequent PIK3CA mutations and recurrent 9p24.1 amplification involving PD-L1, PD-L2, and JAK2, providing a biological rationale for immune checkpoint blockade and other targeted therapeutic strategies (2). Clinically, EBV positivity is associated with a favorable prognosis. A large international pooled analysis using EBER-ISH reported that EBV-positive GC was associated with superior overall survival after adjusting for stage and other prognostic variables (51). Therefore, EBV represents both a molecular subtype with distinct tumor biology and a clinically relevant biomarker that may inform prognosis and therapeutic stratification.

EBV-positive GCs are traditionally diagnosed using EBER-ISH. Multiple H&E-based EBV predictors frame their primary role as prescreening to prioritize confirmatory testing (17, 22). Several studies have attempted to improve the clinical realism of EBV prediction by incorporating human–AI interactions, sequential workflows and cohort-specific fine-tuning. Zheng et al. developed an EBV prediction pipeline with uncertainty-weighted human-AI fusion, achieving strong discrimination on internal evaluation and multicenter external cohorts (AUROCs 0.969 and 0.941, respectively). Importantly, the fusion strategy improved performance beyond that of either the model or pathologist alone, reflecting a clinically realistic decision support paradigm (27). Jeong et al. similarly reported a sequential two-stage EBV prediction pipeline and showed that fine-tuning on an EBER-ISH-labeled cohort improved performance in an external cohort (external AUROC 0.88; area under the precision-recall curve [AUPRC] 0.65; sensitivity 0.86; specificity 0.92 after fine-tuning) (22). The improvement after fine-tuning suggests that EBV prediction may be particularly sensitive to domain shifts and local cohort characteristics. This finding is consistent with the low prevalence of EBV-positive GC and the local histological variations. The biological interpretability of predictions has also been explored. Zhang et al. performed a TCGA-based analysis linking EBV prediction to spatial immune infiltration patterns and showed that EBV model scores correlated with lymphocyte-rich morphology. The study further suggested that model-derived EBV scores may provide prognostic information beyond simple lymphocyte metrics in TCGA dataset (19).

Collectively, the current evidence suggests that H&E-based EBV prediction is biologically plausible and increasingly technically feasible. However, EBV prediction is highly sensitive to class imbalance and cohort-specific morphology; therefore, local calibration of prescreening thresholds and multicenter validation are critical.

### 3.3 GS and CIN

Compared with EBV and MSI, GS and CIN are generally more difficult to approximate using surrogate markers. GS tumors are characterized by the relative absence of extensive copy number alterations and are enriched in diffuse-type morphology and alterations, such as CDH1, RHOA, or CLDN18–ARHGAP fusions. In contrast, CIN tumors are characterized by broad aneuploidy and recurrent copy number alterations, including receptor tyrosine kinase amplification (2). GS and CIN classifications depend more directly on genome-wide or broad copy number profiling. Flinner et al. demonstrated that surrogate approaches for distinguishing GS from CIN GCs, based on Laurén morphology and IHC results of E-cadherin and p53, were discordant with genomic assessment results for CIN performed using OncoScan copy number variation arrays and calculated CIN ratios. Although current models remain limited by label noise, intratumoral heterogeneity, and imperfect molecular ground truths, H&E-based deep learning may provide a scalable prescreening layer that outperforms conventional morphology- and IHC-based surrogate classifications (21). Overall, GS/CIN classification is the most label-sensitive component of TCGA subtyping and requires validation against robust genomic endpoints.

## 4 Molecular alterations and targeted therapy biomarkers

### 4.1 HER2 (ERBB2)

HER2 overexpression or amplification defines a clinically actionable subset of advanced GC or gastroesophageal junction cancer, with trastuzumab plus chemotherapy established as the standard first-line therapy for HER2-positive GC (52). Trastuzumab deruxtecan has demonstrated significantly improved overall survival in patients with HER2-positive metastatic GC (53). Currently, HER2 status is determined using standardized IHC and confirmatory ISH as appropriate, reflecting the threshold-dependent nature of the companion diagnostic paradigm.

Limited access to confirmatory ISH testing can delay HER2 evaluation in a substantial proportion of patients with GC. In this context, HER2Net, a WSI-based deep learning model, infers HER2 status directly from routine H&E-stained slides. HER2Net could serve as a potential alternative to conventional HER2 workflows in resource-constrained settings or, more conservatively, as a decision-support tool to prioritize and contextualize downstream HER2 testing. The interpretation of HER2 IHC remains challenging because of the inter-observer variability among pathologists. By generating spatially resolved predictions of HER2-high regions and quantifying the corresponding area fraction at the pixel-level resolution, HER2Net may improve interpretability and reproducibility, providing objective supportive evidence alongside standard IHC/ISH-based assessments (38).

However, HER2 inference from H&E is particularly sensitive to labeling variability and domain shifts because the clinical reference standard is thresholded and assay-dependent. Furthermore, specific histological patterns influence diagnostic accuracy; adenoid or papillary differentiation is a frequent driver of false-positive predictions, whereas poor differentiation and interstitial fibrosis often contribute to false-negative results (38). Recognizing these morphological and technical boundaries is essential for the safe integration of HER2-predictive AI into pathological workflows.

### 4.2 Other genetic alterations

At the single-gene level, image-based mutation prediction demonstrates both feasibility and limited portability. Jang et al. developed fully automated H&E pipelines to predict multiple genetic alterations in GC, achieving AUROC values of 0.778 (CDH1), 0.833 (ERBB2), 0.838 (KRAS), 0.761 (PIK3CA), and 0.775 (TP53). However, the study showed that direct external generalization can be modest, and cross-cancer transfer may fail, reflecting organ- and cohort-specific morphology–genotype relationships (54). Pan-cancer screening efforts extend this concept by presenting general workflows for the detection of clinically actionable genetic alterations and expression signatures in histopathology. For GC, these results are best interpreted as enabling platforms for triage and hypothesis generation rather than immediate substitutes for sequencing (7). External portability remains a primary barrier to mutation prediction, highlighting the need for multicenter validation and careful deployment planning.

## 5 Immuno-oncology: TMB, PD-L1, and TME

### 5.1 TMB and PD-L1 expression

In GC, PD-L1 expression and TMB/MSI-related immunogenicity are widely used to assess the potential benefits of immune checkpoint inhibitor therapy. TMB is conceptually aligned with MSI/MMR-deficient as a surrogate for tumor neoantigen load and has emerged as a tissue-agnostic biomarker for PD-1 blockade. In June 2020, the U.S. Food and Drug Administration granted accelerated approval for pembrolizumab in patients with unresectable or metastatic TMB-high (TMB-H; ≥10 mutations/Mb) solid tumors (55).

Recent computational pathology studies have attempted to infer TMB and MSI-related immunogenicity directly from routine H&E WSIs. Zhang et al. proposed a dual-branch, cell-aware WSI framework that combined tissue-level image features with nuclear segmentation-derived cellular features. In this framework, CLAM-derived image features and Hover-Net-derived nuclear segmentation features were fused using multimodal compact bilinear pooling and applied to MSI and TMB predictions in TCGA gastric and colorectal cancer datasets. In GC, the best-performing fusion model achieved AUC of 0.835 and 0.800 for MSI and TMB predictions, respectively. In an independent colorectal cancer validation cohort, nuclear feature fusion improved MSI prediction from an AUC of 0.61 using image features alone to 0.81 after fusion (56). These findings suggest that nuclear morphology and cellular composition provide complementary information to conventional WSI-level image features and may improve the biological interpretability of immunotherapy-related biomarker prediction. Nevertheless, as external validation was performed in colorectal cancer rather than GC, GC-specific multicenter validation remains necessary.

PD-L1 functions as an immune checkpoint ligand that suppresses T cell activity via programmed cell death 1 signaling. PD-L1 IHC is routinely used to guide immune checkpoint inhibitor selection in patients with GC. PD-L1 expression is commonly assessed using the combined positive score (CPS), which integrates PD-L1–positive tumor and immune cells and is emphasized as a clinically practical scoring system (57, 58). Several studies have demonstrated the feasibility of inferring PD-L1 status directly from routine H&E WSIs, supporting a prescreening/triage role to prioritize confirmatory PD-L1 IHC (59, 60). The automation of PD-L1 CPS assessment on IHC slides has also been developed to improve reproducibility and scalability (61).

### 5.2 TME architecture

There has been a significant focus on TME-specific analyses to understand tumor behavior and its responses to treatment. In routine practice, molecular phenotyping is often performed using limited tissue regions, despite intratumoral heterogeneity and spatially variable immune architecture. In contrast, WSI preserves spatial context, enabling quantitative analysis of TME components, such as tumor–immune interfaces, stromal patterns, and spatial distribution of lymphocytes and other immune subsets, thereby providing additional prognostic and predictive information relevant to immunotherapies and combination strategies (62, 63).

Approaches that quantify the tumor–stroma ratio (TSR) and tumor-infiltrating lymphocytes (TIL) from H&E suggest that WSIs contain prognostically relevant information beyond tumor cell morphology alone. TMEPATH provides a representative example by integrating TSR and TIL into a composite risk score. This approach stratified patients with GC into prognostic risk groups, was validated in an independent cohort, and was associated with molecular features, including MSI, TMB, and CDH1 mutation status (37). These findings support the concept that computational pathology can convert conventional H&E slides into quantitative TME readouts that complement established clinicopathological and molecular variables.

Spatial immune phenotyping extends this concept from risk stratification to biologically interpretable immune architecture. Pomponio et al. integrated AI image analysis with transcriptome profiling to classify tumors into immune-inflamed, -excluded, and -desert phenotypes by quantifying the spatial distribution of CD8^+^ T cells relative to tumor and stromal compartments using multiplex PanCK/CD3/CD8 immunofluorescence. In the subset with gene expression profiling, the excluded and desert phenotypes were associated with higher TGFβ pathway activation, providing a mechanistic link between the image-derived spatial immune architecture and a potentially targetable immunosuppressive pathway. Collectively, this study supports a TME-centric role for computational pathology as an enrichment tool to identify patients who may benefit from combination strategies aimed at overcoming TGFβ-driven immune exclusion (24). Tian et al. developed a WSI-based “DeepRisk” signature that stratified prognosis in GC and was biologically anchored to an immunosuppressive TME. The high-risk signature was associated with an invasive-margin niche enriched for CD11b^+^CD11c^+^ myeloid/MDSC-like cells, reduced B-cell signals, and increased markers of immune exhaustion, as supported by multiplex IHC and TCGA transcriptomic deconvolution (36). Collectively, these studies position WSI-derived risk models as prognostic tools and TME readouts that can inform hypotheses for myeloid-targeted or combination immunotherapy strategies in GC.

### 5.3 Immune-signature subtypes

Immune architecture can be captured using lymphoid structure-based phenotyping. A large multicohort study developed an interpretable H&E pipeline to detect and classify tertiary lymphoid structures (TLS) and derived a quantitative TLS score that stratified survival risk across multiple gastrointestinal cancer cohorts (29). A mechanistically oriented study further advanced this concept by grading TLS maturity based on H&E staining and integrating orthogonal immune profiling (e.g., multiplex IHC and single-cell RNA sequencing) to connect TLS maturation with specific immune programs, positioning TLS maturity as an interpretable immune-architecture biomarker (40).

A complementary strategy is to infer transcriptome-derived immune infiltration patterns from H&E images. Han et al. developed a machine-learning pathomics model using TCGA GC H&E slides, transcriptome-based immune deconvolution, PyRadiomics feature extraction, and a gradient-boosting machine classifier to predict activated CD4 memory T cell infiltration. The resulting pathomics score was independently associated with improved overall survival and was linked to antigen processing and presentation, interferon-related pathways, CD8 T-cell and M1 macrophage infiltration, and higher TMB, suggesting that H&E-derived pathomics can capture immune-active TME biology beyond tumor cell morphology. However, because the immune cell labels were inferred from transcriptomic deconvolution rather than direct spatial immune cell measurements, further validation using multiplex IHC or spatial profiling would strengthen their biological and clinical relevance.

A complementary immune-surrogate approach extends H&E-based risk stratification by predicting transcriptome-derived immune cell infiltration patterns using routine histology. In this framework, a machine-learning pathomics model estimated activated CD4^+^ memory T-cell infiltration, and the predicted immune surrogate was independently associated with overall survival after covariate adjustment, linking morphological features to an immune-related prognostic axis of survival (34).

## 6 Multimodal integration

Multimodal models are motivated by the complementary nature of clinical data streams. Radiology captures macroscopic tumor burden, anatomical extent, and spatial heterogeneity; pathology captures cellular morphology and TME; omics provides direct measurement of molecular states; and clinical variables are contextualized. The key message emerging from the current studies is that adding more data improves performance, but that multimodal AI is most useful when different modalities provide non-redundant information at the same clinical decision point. In GC, this is particularly relevant because treatment response, recurrence, survival, and metastatic risk are shaped by interactions among tumor morphology, immune microenvironment, molecular programs, anatomical disease extent, and patient-level clinical factors.

Multimodal integration has been actively explored to predict treatment responses. Gao et al. developed an interpretable incremental supervised contrastive learning model that integrated pretreatment contrast-enhanced CT scans with H&E-stained biopsy WSIs to predict response to neoadjuvant chemotherapy in locally advanced GC. The model achieved AUROCs of 0.866–0.876 in external test cohorts and 0.846 in a prospective cohort, outperforming the CT-only, pathology-only, and direct feature concatenation models. This study suggests that effective multimodal learning requires cross-modal feature alignment rather than simple concatenation. The model also provided biologically interpretable signals: attention was enriched near the tumor-invasive border, and responder-associated regions showed increased inflammatory cell infiltration (42). These findings illustrate how radiology–pathology fusion can link macroscopic tumor extent with microscopic immune architecture relevant to chemotherapy sensitivity.

A related direction is the multimodal prediction of targeted and immunotherapeutic responses. Chen et al. developed MuMo, a transformer-based multimodal model for HER2-positive GC that integrated CT images, WSIs, structured radiology and pathology reports, and patient-level clinical information. The model achieved AUCs of 0.821 for anti-HER2 therapy and 0.914 for anti-HER2 therapy combined with anti-PD-1/PD-L1 immunotherapy, and MuMo-defined low-risk patients showed significantly longer overall and progression-free survival (43). A major strength of this study is its explicit accommodation of incomplete real-world multimodal data. By representing unavailable radiologic or pathologic inputs with learnable modality-specific placeholders, the model enabled multimodal training and inference even when not all input modalities were available for each patient. Ablation analyses further showed that intermodal fusion and modal-agnostic feature alignment outperformed the simple concatenation. Therefore, this study highlights a practical principle for clinical use: models should be designed not only for ideal complete datasets but also for incomplete and asynchronous data streams that are typical in routine oncology settings.

For postoperative recurrence prediction, Ding et al. provided one of the strongest examples of clinically oriented multimodal integration in GC. Their Recurrence Stratification Assessment (RSA) model combined clinical predictors, CT radiomics, and H&E-derived pathomics using an interpretable score-level fusion strategy. In 1,580 patients with locally advanced GC from six Chinese centers, the model outperformed single-modality models. Transcriptomic analyses have linked high-risk predictions to immune cell infiltration, immune checkpoint expression, interferon signaling, and IL-6/JAK/STAT3 pathway activation, supporting the biological relevance of multimodal recurrence risk (11). However, its reduced performance after neoadjuvant chemotherapy indicates that treatment-specific domain shifts are major barriers.

Several studies have directly compared histopathology-only models with multimodal approaches that integrate histopathology-derived risk scores with clinical and gene expression variables, reporting improved discrimination when multimodal information is incorporated. For survival prediction, these approaches generally yield modest but consistent improvements (32, 45, 48). Wei et al. developed an H&E-derived survival risk score was combined with age and selected gene expression features. The multimodal model improved the C-index from 0.660 for the histology-only model to 0.744 when clinical and gene expression data were incorporated, supporting the complementary prognostic value of tissue morphology, patient-level variables, and transcriptomic programs (32). Xie et al. further developed a cross-modal attention framework that integrates WSIs with pathway-selected gene expression features for survival prediction, pathological stage classification, and lymph node classification (45). Their interpretability analysis suggested that WSI features dominated many predictions, whereas invasion- and metastasis-related gene expression substantially contributed to the selected cases. These studies support the biological rationale for histology–omics fusion; however, their clinical readiness remains limited by small cohorts, external validation, and the fact that transcriptomic data are not routinely available for all GC patients.

The magnitude of improvement from multimodal integration depends on the task. For genomics-defined targets, such as TMB, the addition of omics data can substantially improve predictive performance because omics data directly measure molecular states. Li et al. showed that an H&E-only ResNet18 model predicted TMB status in TCGA GC with an AUC of 0.749, whereas H&E–mRNA fusion using multimodal compact bilinear pooling increased the AUC to 0.971 (44). For lymph node metastasis prediction, Guo et al. developed a multimodal multiscale model that integrated WSIs at 10×, 20×, and 40× magnifications with clinical variables, including age, sex, Lauren classification, and tumor location. The model achieved a mean AUC of 0.877 in cross-validation using radical surgery slides and outperformed single-scale and clinical-only models. However, performance decreased when evaluated on preoperative biopsy slides, with a mean AUC of 0.725, and further declined in TCGA-based evaluation after transfer learning (41). When the purpose is preoperative decision-making, clinical translation is challenging because the highest performance is obtained using resection specimens. This highlights a central limitation of computational pathology models: validation must match the specimen type, tissue quantity, and timing of the intended clinical application.

## 7 Discussion

### 7.1 Interpretability, explainability, and biological plausibility

Interpretability is essential for clinical trust and adoption because pathologists and oncologists must determine whether the model predictions are biologically plausible, clinically actionable, and safe to integrate into patient care. In computational pathology, interpretability should not be limited to attention heatmaps, tile-level exemplars of biomarker-like regions. Rather, it should establish a connection between the model outputs, recognizable histologic structures, and clinically meaningful biology. Quantitative immune-architecture readouts, such as TLS, provide spatially grounded explanations (29, 37, 40).

Biological triangulation further strengthens model credibility, such as image-derived predictions supported by orthogonal molecular, immunological, or spatial evidence. For example, immune phenotyping linked to transcriptomic pathway activity, such as TGF-β signaling, provides a mechanistic link between spatial morphology and immunosuppressive biology (24). Similarly, multiplex IHC, single-cell or bulk transcriptomics, and spatial immune validation can help determine whether H&E -derived signatures reflect genuine tumor biology rather than spurious technical correlates (36, 40). Such validation is particularly important for biomarker prediction tasks because high discrimination alone does not prove whether a model learns the intended molecular phenotype.

Uncertainty estimation provides an additional layer for clinical interpretation. In biomarker triage workflows, an AI model should ideally identify cases in which its prediction is unreliable and redirect them for expert review or confirmatory testing rather than forcing an overconfident binary output (39). This is especially relevant for MSI, EBV, HER2, and PD-L1-related predictions, where intermediate or uncertain cases may be driven by limited tumor content, poor tissue quality, mixed histology, treatment effects or spatial heterogeneity. Human–machine integration further operationalizes interpretability by combining model-derived probability and confidence with pathologists’ judgment. Therefore, the most clinically realistic framework is not autonomous diagnosis but pathologist-supervised decision support, in which AI highlights relevant regions, estimates probability, flags uncertainty, and helps prioritize downstream testing (27).

### 7.2 Limitations, clinical translation, and future directions

Despite promising performance, most studies remain retrospective, and real-world performance may differ substantially because of variability in tissue processing, staining protocols, scanner characteristics, image quality, molecular testing practices, and patient population. Even in multicohort studies, performance often varies across datasets, indicating that domain shift remains one of the most persistent barriers to clinical translation (17, 28). This issue is particularly relevant in GC, where histologic heterogeneity, biopsy-versus-resection differences, treatment-induced changes, and geographic variations in molecular subtype prevalence may affect the robustness of the model.

The methodological limitations of this field are not trivial. A recent systematic review of machine learning and deep learning applications in GC found that external validation was absent in approximately two-thirds of studies, explainability techniques were not implemented in many studies, and dataset partitioning was often reported incompletely (64). These findings reinforce the fact that many AI models in GC remain closer to technical feasibility than clinical maturity. Therefore, future studies should routinely include site-held-out validation, transparent quality control procedures, calibration assessment, uncertainty estimation, pre-specified clinical operating points, and reporting standards, such as CLAIM. Global performance values alone are insufficient because clinically useful models must demonstrate stable performance based on decision-relevant thresholds.

Clinical translation also requires clarity on the intended use. The threshold and validation strategies for an intended model should differ depending on the purpose, such as a rule-in prescreening tool, treatment response predictor, or prognostic risk stratifier. For example, an MSI or EBV prescreening model may prioritize sensitivity and negative predictive value to reduce unnecessary confirmatory tests, whereas a model used to select patients for neoadjuvant or adjuvant treatment must demonstrate calibration, clinical net benefit, and robustness across all treatment regimens. Prospective studies should evaluate not only discrimination but also whether AI improves turnaround time, testing efficiency, resource utilization, treatment selection, surveillance strategy, patient outcomes, and cost-effectiveness (65).

Several practical barriers must be addressed before multimodal AI can be routinely adopted for GC care. First, complete multimodal datasets are uncommon. A patient may have diagnostic biopsy slides but no resection specimens, baseline CT but no standardized response annotation, or clinical variables collected at different time points. Restricting model development to cases with all modalities available can reduce the sample size and introduce selection bias. Second, missingness is random; it may reflect the disease stage, institutional practice, insurance coverage, tissue availability, treatment intent, or patient selection for molecular testing. Therefore, future models should explicitly report missing data patterns and incorporate missing modality strategies, such as modality dropout, uncertainty-aware prediction, or learnable placeholder representations (11, 43).

Third, harmonization remains a major challenge in this field. Pathology images vary according to fixation, staining, section thickness, scanner type, magnification, compression, and tissue artifacts. Radiologic images vary according to the acquisition protocol, contrast phase, reconstruction parameters, segmentation strategy, and selection of lesions. Omics data vary by platform, panel design, sequencing depth, normalization, and bioinformatic pipelines. Clinical variables are often inconsistently recorded across different institutions. Without standardized preprocessing and transparent reporting, multimodal models may learn site-specific technical signatures rather than disease biology. Fourth, temporal alignment is essential. Models that combine biopsy pathology, baseline CT, post-treatment imaging, resection pathology, and molecular profiling must consider the time at which each modality is acquired. In addition, data input regarding the timing of treatment is significant because therapy can alter both tissue morphology and imaging phenotype.

The interpretability and clinical utility of multimodal models should be evaluated beyond global performance metrics. A higher AUROC or C-index does not necessarily indicate that all modalities contribute to meaningful biological information. Multimodal studies should include modality-ablation analyses, calibration, decision curve analysis, subgroup validation by specimen type and treatment setting, and assessment of whether the model improves decisions beyond the existing clinicopathologic and molecular standards. These analyses are necessary to determine whether each modality adds complementary biological information or merely introduces confounding factors related to stage, treatment selection, or institutional workflow.

Workflow integration and governance are equally important for the clinical adoption of AI. Professional guidance from the College of American Pathologists emphasizes that AI tools should complement and augment pathologists’ diagnostic capabilities rather than replace physician expertise (65). This principle is directly relevant to GC biomarker prediction, where AI outputs are probabilistic and often require confirmatory molecular or immunohistochemical testing. Regulatory frameworks further reinforce the need for risk-based AI governance. The European Commission proposal for the AI Act emphasizes data governance, technical documentation, traceability, transparency, accuracy, human oversight, cybersecurity, robustness, and post-market monitoring of higher-risk AI systems (66). Similarly, the Korean MFDS guidance for machine learning-enabled medical devices highlights independent validation datasets, clinically interpretable performance metrics, reference standard definitions, documentation of data collection and inclusion criteria, cybersecurity, version control, change approval, and training dataset management (67).

Finally, data-sharing constraints remain a major obstacle to the development of robust models. Multinational collaborative training highlights the importance of cohort diversity for generalizability, whereas decentralized or privacy-preserving learning approaches suggest that performance comparable to centralized training may be achievable without the direct sharing of raw patient data (17, 30). Such strategies may be particularly important for rare biomarker-defined subgroups or treatment-response cohorts, where single institutions are unlikely to accumulate sufficiently large and diverse datasets.

## 8 Conclusion

AI-based biomarker prediction in GC is moving from technical proof-of-concept to clinically oriented decision support. The near-term role of computational pathology is most likely to be in prescreening, triage, risk stratification, treatment response enrichment, and spatial interpretation. Future studies should be subject to prospective multicenter validation, standardized reporting, revealing clinically calibrated thresholds, handling domain shifts, managing missing modalities, local workflow integration, and establishing evidence that AI improves patient management beyond current standard-of-care approaches.
